# Vitamin A in Skin and Hair: An Update

**DOI:** 10.3390/nu14142952

**Published:** 2022-07-19

**Authors:** Christine A. VanBuren, Helen B. Everts

**Affiliations:** Department of Nutrition and Food Sciences, Texas Woman’s University, Denton, TX 76209, USA; cvanburen@twu.edu

**Keywords:** vitamin A, hair follicle stem cells, melanocyte stem cells, melanocyte, retinoids

## Abstract

Vitamin A is a fat-soluble micronutrient necessary for the growth of healthy skin and hair. However, both too little and too much vitamin A has deleterious effects. Retinoic acid and retinal are the main active metabolites of vitamin A. Retinoic acid dose-dependently regulates hair follicle stem cells, influencing the functioning of the hair cycle, wound healing, and melanocyte stem cells. Retinoic acid also influences melanocyte differentiation and proliferation in a dose-dependent and temporal manner. Levels of retinoids decline when exposed to ultraviolet irradiation in the skin. Retinal is necessary for the phototransduction cascade that initiates melanogenesis but the source of that retinal is currently unknown. This review discusses new research on retinoids and their effects on the skin and hair.

## 1. Introduction

Adequate consumption of vitamin A is necessary for the maintenance of healthy skin and hair. This is because vitamin A affects the skin and hair in a dose-dependent manner, where too much or too little has deleterious effects. During vitamin A deficiency, follicular hyperkeratosis develops, which is resolved with large doses of vitamin A [1]. Vitamin A deficiency causes changes in epithelial tissues, replacing simple epithelial cells with stratified keratinizing epithelium [2]. Excess vitamin A consumption, through use of retinoid treatments or excessive vitamin A supplementation, also results in hair loss and reduced sebaceous gland function, albeit through a different mechanism than follicular hyperkeratosis [3]. This reflects the hormesis effect of vitamin A consumption [4]. As the intake of vitamin A increases, more retinoic acid (RA) is produced and provides a beneficial effect up to a point. When that level is reached, however, vitamin A supplementation becomes toxic and disrupts the proper functioning of the organism. The purpose of this review is to provide an update of the latest findings on the role of retinoids in skin and hair. Specifically, we will discuss the regulation of hair follicle stem cells (HFSCs) through dietary vitamin A intake, retinoid metabolism in the melanocyte, and the effects that ultraviolet irradiation has on retinoid metabolism.

## 2. Vitamin A Metabolism

Retinol travels through the bloodstream bound to retinol binding protein 4 (RBP4) and transthyretin (prealbumin) [5]. Upon reaching its target tissues, retinol enters the cell through passive diffusion or through one of two multi-transmembrane receptors, stimulated by retinoic acid 6 (STRA6) or retinol binding protein receptor 2 (RBPR2) [6,7,8]. As retinol passes through its receptor, it binds to cellular retinol binding protein 1(RBP1) where it is esterified [9] by lecithin:retinol acyltransferase (LRAT) [10] or acyl-CoA:diacylglycerol acyltransferase 1(DGAT1) [11]. This esterification sequesters retinol, both preventing it from leaving the cell and from entering oxidative metabolic pathways [12]. Unesterified retinol is interconvertible with retinal through oxidation/reduction reactions. 

Vitamin A (retinol) is oxidized to retinal through the action of microsomal retinol dehydrogenases (RDH). RDHs function as gatekeepers, limiting the downstream creation of retinoic acid [13]. In the skin and hair follicle, there are five known members of the short-chain dehydrogenase reductase (SDR) superfamily that convert retinol to retinal. These include retinol dehydrogenases 1/16 (RDH1/16) [14,15], RDH 10 [16,17], RDHE2, and RDHE2S [18,19], and dehydrogenase reductases SDR family member 9 (DHRS9) [20,21,22]. DHRS3 is also within this superfamily but prevents toxicity by catalyzing the opposite reaction (retinal to retinol) [23]. Retinal is oxidized irreversibly to RA by retinal dehydrogenases 1–3 (ALDH1A1, ALDH1A2, and ALDH1A3) and then bound to cellular retinoic acid binding protein (CRABP) 1 or 2 [24]. RA bound to CRABP2 is chaperoned to the nucleus and is transferred to retinoic acid receptors (RARA, RARB, and RARG) to regulate transcription [25,26]. CRABP1 can transport RA to the nucleus, but is less efficient than CRABP2. Both CRABP1 and CRABP2 can chaperone RA to the cytochrome 450 enzymes CYP26A1, CYP26B1, and CYP26C1 located in the endoplasmic reticulum to be degraded [24,27].

Retinoic acid receptors (RARs) and retinoid X receptors (RXRs) are nuclear transcription factors that are activated by RA. They are potent regulators of the cell cycle, differentiation, proliferation, and apoptosis [28,29]. In addition, RA can also initiate different signaling pathways. First, the production of RA stimulates the transmembrane receptor STRA6 to localize to the cell membrane, where it catalyzes the entry of retinol into the cell from the transport protein RBP4 [6]. STRA6 is activated when bound to holo-RBP4. This triggers tyrosine phosphorylation on the C-terminal tail, resulting in the recruitment of janus kinase 2 (JAK2) and signal transducer and activator of transcription 3 and 5 (STAT 3/5) [30]. STRA6 has also been shown to participate in p53-mediated apoptosis through the intrinsic pathway following damage to cellular DNA or increased intracellular levels of reactive oxygen [31]. As a retinoic acid-responsive gene, ***Stra6*** may play an important role in the maintenance of healthy skin and hair. While not extensively studied, ***Stra6*** has been shown to be decreased in C3H/HeJ mice with spontaneous alopecia areata [32]. It has also been shown to be upregulated in mice with skin lesions in a mouse model of psoriasis [33] Interestingly, a study examining the effects of STRA6 knockdown in both epidermal keratinocytes and a human skin model found that the knockdown of STRA6 resulted in increased epidermal proliferation and epidermal thickening [34]. Second, excess RA can bind fatty acid binding protein 5 (FABP5), activate the peroxisome proliferator-activated receptor B/D (PPARB/D), and initiate transcription of a different set of genes [35].

## 3. Hair Follicle Stem Cells (HFSCs) and the Hair Cycle

HFSCs regulate the hair cycle and wound healing in normal conditions [36,37], although dysregulation of HFSCs leads to skin cancers [38]. The hair cycle consists of five stages: anagen (hair follicle differentiation and hair shaft growth), catagen (hair follicle regression), refractory telogen (HFSC quiescence), competent telogen (HFSCs ready for activation), and exogen (hair shaft release) [39,40,41]. Bone morphogenetic protein 2, 4, 6 (BMP2, BMP4, BMP6), fibroblast growth factor 18 (FGF18), forkhead box c1 (FOXC1), LIM homeobox protein 2 (LHX2), and nuclear factor of activated T cells 1, cytoplasmic (NFAT1C) inhibit HFSCs to maintain refractory telogen [42,43,44,45,46]. In contrast, WNT7A, WNT7B (wingless-related MMTV integration site 7A and 7B), fibroblast growth factor 7(FGF7), transforming growth factor beta (TGFB), and the BMP inhibitor noggin activate HFSCs and anagen initiation [47,48,49,50,51,52]. 

**Figure 1:**
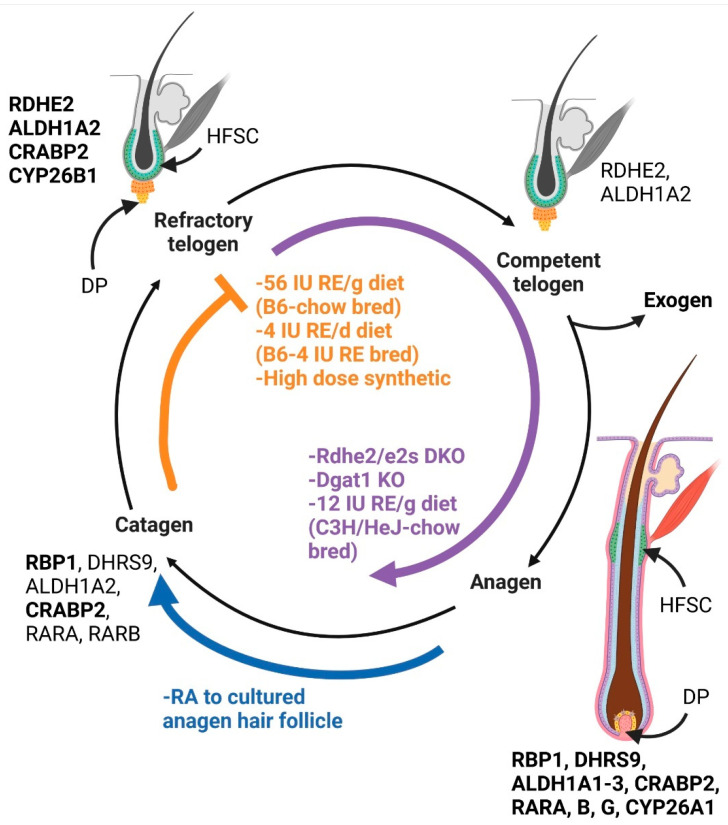
**Retinoid effects on the hair cycle:** The five stages of the hair cycle are indicated. The vitamin A metabolism proteins expressed at that stage in chow fed C57BL/6J (B6) mice are indicated near that stage [20,54]. Bolded proteins are expressed at higher levels than non-bolded proteins. Conditions listed in orange have more hair follicles in refractory telogen. This includes both the highest and lowest levels of dietary vitamin A in B6 mice and high dose synthetic retinoids given to humans [54,57,59]. Conditions in purple have greater numbers of hair follicles or an acceleration of anagen induction. This includes Rdhe2/e2s double null mice (DKO) with reduced retinal, Dgat1 null mice (KO) with excess RA, and C3H/HeJ mice fed moderate levels of vitamin A [19,32,55]. Conditions in blue indicate catagen induction. This includes exogenous RA provided to cultured anagen hair follicles [63,64]. HFSC = hair follicle stem cell, DP = dermal papilla. Created in BioRender.com.

Genetic and dietary studies suggest that RA may regulate HFSCs with a U-shaped dose–response curve (Figure 1). RA synthesis and signaling proteins localized to the hair follicle with increased levels seen during refractory telogen, mid-late anagen, and catagen [20,53,54]. Both reduced RA in *Del(4Sdr16c5-Sdr16c6)1Nyk* (Rdhe2/Rdhe2s) double null mice and excess RA in skin-specific Dgat1 (*Dgat1^tm2Far^Tg(KRT14-cre)1AMC*) null mice led to more hair follicles in anagen when fed a chow diet [19,55]. This lengthened anagen could be caused by lack of HFSC inhibition during refractory telogen, leading to early anagen or lack of catagen induction. Feeding a vitamin A deficient diet partially restored the hair cycle in the *Dgat1^tm1Far^* null mice and worsened the phenotype in the *Del(4Sdr16c5-Sdr16c6)1Nyk* double null mice. Dietary vitamin A altered the hair cycle differently in three studies [32,54]. One study was carried out in C3H/HeJ mice to study alopecia areata, an autoimmune hair loss disease where immune cells attack anagen hair follicles [32]. These mice were fed an unpurified diet during breeding, then fed the AIN93M diet with 0, 4, 12, or 28 IU vitamin A/g diet two weeks before inducing the disease. Increasing the vitamin A in the diet raised the percent of hair follicles in anagen and made the disease worse [32,56]. The next two studies were carried out to test the hypothesis that excess vitamin A worsens central centrifugal cicatricial alopecia (CCCA), a form of permanent hair loss seen primarily in African American women where HFSCs are destroyed [57]. CCCA spontaneously develops in C57BL/6J mice when hair follicles are in anagen [58]. Two feeding studies were conducted on these mice. In study one, female C57BL/6J mice were bred on the unpurified diet and then fed the AIN93M diet with 4, 28, and 56 IU vitamin A/g diet starting at 12 weeks of age for 16 weeks. Feeding mice the highest level of vitamin A resulted in more hair follicles in refractory telogen and less CCCA [54,57]. A follow-up study was conducted to reduce vitamin A levels. In study 2, mice were bred for three generations on the AIN93G diet. At 6 weeks of age, the diet was switched to the AIN93M diet with 4, 28, or 56 IU vitamin A/g diet for 12 weeks. Mice fed the 4 IU vitamin A/g diet in study 2 had more hair follicles in refractory telogen than those fed the highest level [54]. These studies imply that vitamin A regulates the hair cycle, but it depends on dose, timing of the diet change, and/or mouse strain. In addition, pharmacological doses of synthetic retinoids Etretinate and Acitretin increased telogen and hair loss due to telogen effluvium (hair loss caused by more hair follicles entering telogen and loss of anchorage support during telogen) [59]. Blocking RA degradation with *Cyp26b1^tm1Hh^* null mice impaired embryonic hair development and reduced *Lhx2*; but these mice died before the hair cycle could be investigated [60]. RA also regulates the differentiation of both human embryonic [61] and mouse-induced pluripotent stem cells to differentiate into keratinocytes in vitro, which upon transplantation to nude mice develop fully functional skin [62]. This regulation requires both precise doses and application of RA at specific times during differentiation [61].

RA may also regulate the anagen to catagen transition. Catagen is a state of massive apoptosis that results in the shrinking of the hair follicle [39]. Studies in both cultured human and mink hair follicles found that RA dose-dependently increased catagen induction through activation of the TGFB2/smad2/3 pathway [63,64]

In summary, these studies suggest that RA regulates both anagen and catagen induction. RA may inhibit HFSCs to keep them in refractory telogen. In addition, RA may trigger catagen. Both of these functions would lead to a longer anagen if RA were to be limited, as seen in genetic studies of retinoid metabolism genes. Future studies should examine the molecular mechanisms of these effects. 

## 4. Wound Induced Hair Follicle Neogenesis (WIHN)

WIHN is a process where new hair follicles are formed within a healing wound if the wound is large and deep enough [65]. This wound healing process is preferred because other types of wound healing leads to scars. Kim et al. (2019) found that such large wounds triggered endogenous RA synthesis [66]. More specifically, large wounds increased noncoding dsRNA, which increased ALDH1A3, RARA, and RA levels via the toll-like receptor 3 (TLR3) signaling pathway. In addition, inhibiting ALDH1A3 or RARA blocked WIHN. Kim et al. (2019) found that laser treatments also increased RA levels in human skin, suggesting that RA may be involved in several damage responses. Induction of these damage responses are used cosmetically to make skin look young. Abbasi et al. (2020) confirmed this role of RA in WIHN and found that CRABP1 positive upper dermal fibroblasts were required [67]. They found numerous genes and accessible chromatin enhanced in the CRABP1 positive upper dermal fibroblasts, but did not directly determine the genes altered by RA. These upper dermal fibroblasts are predicted to develop into the dermal condensate, which sends signals to the epidermis to initiate the formation of new hair follicles, similar to embryonic development. Phan et al. (2021) determined that these CRABP1 positive upper dermal fibroblasts became dermal papilla cells in the presence of sonic hedgehog [68]. The dermal papilla is a collection of mesenchymal cells that sits below HFSCs in telogen hair follicles and sends signals to regulate these HFSCs [3]. These signals include WNT7A, WNT7B, Noggin, and FGF7, which all activate anagen.

## 5. Melanocytes

Retinoids may also be important in melanocyte differentiation. Melanocytes derive from neural crest cells during embryogenesis [69]. Melanocyte stem cells (McSCs) localize to the same sites as HFSCs, the hair follicle bulge area [70]. In one study, genetic deletion of the Notch repressor recombination signal-binding protein Jκ (RBP-J) in HFSCs in vivo led to increased *Sdr16c5*, *Rdh1*, *Rdh9*, *Crabp2*, *Fabp5*, and endogenous RA [71]. This increased RA led to ectopic melanocyte differentiation via c-Kit, as confirmed with both RA synthesis inhibition (WIN 18446) and topical RA. 

**Figure 2:**
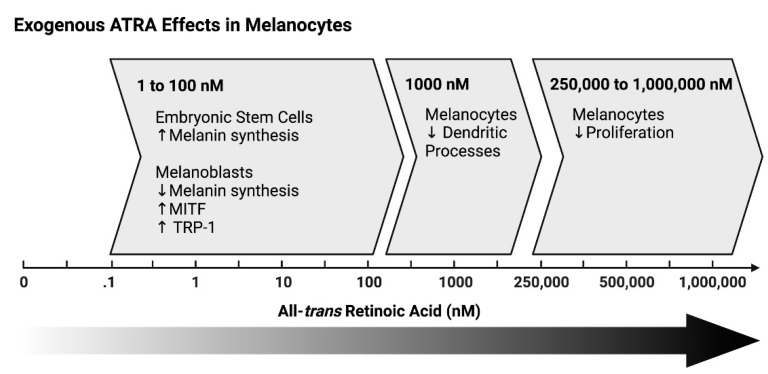
Dose-dependent effects of exogenous RA on melanocytes: 01–100 nM: In embryonic stem cells, ATRA promoted melanin synthesis into melanoblasts. In melanoblasts, ATRA inhibited melanin synthesis [74]. In melanoblasts, ATRA increased both melanocyte-inducing transcription factor and tyrosine-related protein 1, but inhibited these genes in melanocytes [75]. After 12 h of 1000 nM ATRA, melanocytes started to lose dendritic processes [72]. Proliferation was reduced at 250,000 nm ATRA and stopped at 1,000,000 nm. Melanocyte dendrites were lost [73]. Created in BioRender.com.

Variable results are seen in cultured melanocytes treated with RA (Figure 2). In studies, all-*trans* RA increased, decreased, or had no effect on melanogenesis based on specific experimental conditions [72,73,74,75,76]. The addition of physiological doses of RA to cultured murine embryonic stem cells increased melanocyte differentiation only when applied early during this process (before melanoblasts formed) or throughout the process [74]. In contrast, RA reduced melanocyte differentiation if only provided after the melanoblasts formed. Similarly, Kawakami et al. found that physiological levels of all-*trans* RA increased melanocyte inducing transcription factor (MITF) and tyrosinase related protein 1 (TRP-1) when treating melanoblasts, but reduced these genes in melanocytes [75]. In addition, all-*trans* RA dose-dependently increased melanin amounts with a peak at the 6-h time point [72]. Furthermore, treating cultured human mature melanocytes with all-*trans* RA inhibited their proliferation and caused them to lose their dendritic processes [72,73]. These melanocytes became darker and flat in their appearance. When RA was removed, the melanocytes reverted back to their previous state before treatment with RA. Romero et al. (1994) found that pharmacological RA (13-*cis* and all-*trans*) halted UVB-stimulated melanin synthesis and decreased the expression and activity of tyrosinase [77]. In contrast, ALDH1A1 and 9-*cis* RA increased MITF and tyrosinase (TYR) message levels in cultured melanocytes [78]. Paterson et al. (2013) found that 9-*cis* retinal also increased these genes, but not when ALDH1A1 was absent, suggesting that this is due to the 9-*cis* RA and not 9-*cis* retinal [78].

These studies suggest that RA synthesized in keratinocytes in vivo induced differentiation of McSCs to form melanocytes. In vitro, exogenous RA only induced differentiation if provided early. Melanocytes are capable of synthesizing RA as well. Treating mature melanocytes with all-*trans* RA, however, impairs their differentiation in culture. 

RA synthesis proteins have not been extensively examined in melanocytes. RPE65 is expressed in melanocytes, however, lecithin: retinol acyltransferase (LRAT) is not normally expressed at the protein level in melanocytes [79]. *Stra6* was barely detectable at the message level in cultured primary human melanocytes in one study [34]. *Aldh1a1* is critical to melanocytes, as depleting *Aldh1a1* has been shown to impair melanogenesis [78]. In contrast, another study found that CRBP, DHRS9, ALDH1A3, and RARA localized to the premedulla/precortex area of the anagen hair follicle where the hair fiber is initially differentiating and acquiring melanin from melanocytes [20]. It is not clear from this study whether these proteins are expressed within the keratinocytes or melanocytes in this area. Future studies are needed to determine the specific retinoid metabolons that are present in the melanocyte. 

## 6. The role of Retinal in Epidermal Skin Cell Phototransduction

**Figure 3:**
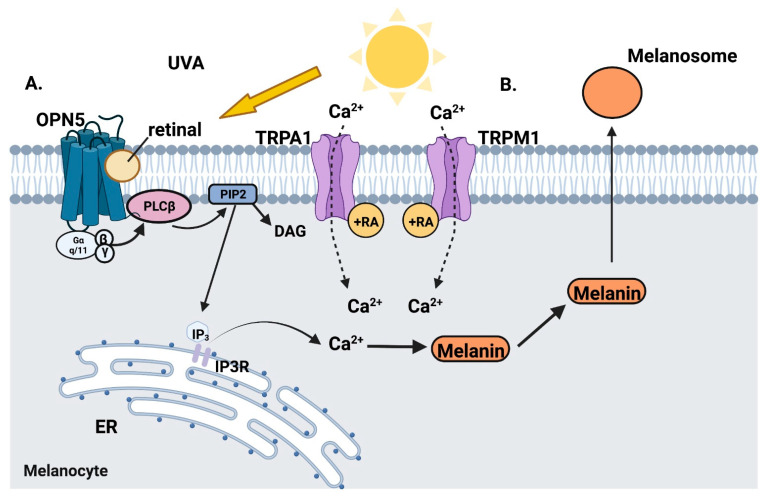
Retinal and melanogenesis: Pictured above are two retinal-dependent processes involved with melanogenesis. A. UVA stimulates retinal-dependent neuropsin (OPN5), which is a g-protein coupled receptor [82,83,84]. Phospholipase Cβ (PLCβ) is activated, which then cleaves phosphatidylinositol 4,5 bisphosphate into two parts: diacylglycerol and inositol triphosphate (IP3) [84]. IP3 binds to its receptor IP3R, located on the endoplasmic reticulum, where it triggers the release of calcium [84]. TRPA1 is then stimulated, bringing extracellular calcium into the cell to facilitate melanin synthesis [81]. B. After melanogenesis, melanosomes are transferred from melanocytes to keratinocytes in a retinal-dependent manner [88]. In response to an increase in microphthalmia-associated transcription factor, TRPM1 is activated. Hu et al. observed that a single dose of UVA (3 J/cm^2^) caused a quick uptake of Ca^2+^ into the melanocyte and melanosome transfer if retinal was present. UVB (20 mJ/cm^2^) resulted in a later (10–30 min) retinal-dependent uptake of calcium into the melanocyte and melanosome transfer. Created in BioRender.com.

Phototransduction by epidermal opsins in response to ultraviolet irradiation performs key processes in the regulation of melanogenesis (Figure 3). Opsins are g-protein coupled receptors that are light-sensitive [80] and require two parts to be functional: the opsin apoprotein and a retinal chromophore [80]. There are four opsins (OPN) known to be expressed in both epidermal melanocytes and keratinocytes: OPN1-SW (cone opsin–short wavelength), OPN2 (rhodopsin), OPN3 (encephalopsin), and OPN5 (neuropsin; [80,81].

In the melanocyte, ultraviolet A (UVA) radiation activates OPN5 [82]. This reaction is retinal-dependent [83] and leads to the activation of g-protein coupled receptors Gαq/11 [84]. Phospholipase Cβ is stimulated and cleaves phosphatidylinositol 4,5-bisphosphate (PIP2) through hydrolysis into diacylglycerol (DAG) and inositol triphosphate (IP3) [84]. IP3 binds its receptor IP3R on the endoplasmic reticulum where it mediates the release of calcium into the cytosol, increasing intracellular calcium levels [84]. Additional calcium is released through the activation of transient receptor potential A1 (TRPA1) ion channels on the plasma membrane, which is necessary for rapid melanin synthesis [81].

Another retinal-dependent Ca channel, transient receptor potential cation channel subfamily M member 1 (TRPM1), is expressed mainly in melanin-producing cells and is abundant in human epidermal melanocytes [85]. TRPM1 transcription is directly increased by microphthalmia-associated transcription factor (MITF) [86,87]. Hu et al. (2017) found that after melanin synthesis, melanosomes are transferred through melanocyte dendrites to keratinocytes in a retinal and TRPM1 dependent manner [88]. A single dose of UVA (3 J/cm^2^) caused a quick uptake of Ca^2+^ into the melanocyte and melanosome transfer if retinal was present. Alternatively, a single dose of UVB (20 mJ/cm^2^) resulted in a later (10–30 min) retinal-dependent uptake of calcium into the melanocyte and melanosome transfer [88].

In summary, retinal is critical for UV induced melanin synthesis by a mechanism similar to the first steps of phototransduction in the eye. In addition, 9-*cis* RA may also induce the transcription of some of these critical channels and melanin synthesis proteins. However, it is unclear whether melanocytes are synthesizing this retinal or obtaining it from neighboring keratinocytes because cultured melanocytes require the addition of retinal and no studies have looked at retinol dehydrogenases in melanocytes. Future studies are needed to answer this question. 

## 7. The Effects of UVA and UVB Irradiation on Retinoic Acid in the Epidermis and Epidermal Skin Cells

Studies have shown that UV irradiation reduces both retinol and retinyl ester concentrations in mouse skin according to UV wavelength [89,90,91,92,93]. UVB produced a significant, dose-dependent decrease in both retinol and retinyl esters that plateaued after 200 mJ/cm^2^ [91]. UVA depleted retinol and retinyl esters at a slower rate, but more completely than UVB in another study [90]. Pre-treating the skin with topical all-*trans* retinal before UVB exposure accelerated the recovery of epidermal retinol and retinyl esters post-UVB treatment [93]. The activity of retinyl ester hydrolases and ARAT in the epidermis did not change in response to UVB exposure [91], but RXRA [92], RBP1 [93], and LRAT [91] were reduced. Gressel et al. (2015) also found decreased expression of LRAT in SKH1 hairless mice (Crl:SKH1-***Hr^hr^***) after UVB exposure [94]. However, UVB increased RBP1, DHRS9, and ALDH1A2 in the upper layers of the epidermis and CYP26A1 throughout the epidermis [94].

In cultured keratinocytes and melanocytes, UV irradiation also depleted cellular retinol and RA. Andersson et al. (1999) found that the uptake of RA in non-irradiated cells was significantly higher in melanocytes compared to keratinocytes [95]. Once treated with UVA (360 mJ/cm^2^) and UVB (140 mJ/cm^2^), there was an immediate decline in retinol concentration in both cell types. The decline resolved within 1–2 days when retinol was added to the cultured keratinocytes and melanocytes. Interestingly, the retinol level in the melanocytes increased to 2–3 times the level that was in the keratinocytes.

UV irradiation affects the function of nuclear retinoid receptors. In a study examining the in vivo effects of UVB exposure on human skin, Wang et al. (1999) found that UVB significantly reduced both the mRNA and protein levels of RARG and RXRA [96]. This effect was diminished by pretreating the skin topically with RA before exposure to UVB irradiation, which possibly means that RA may be protective of the RARG and RXRA nuclear receptors. Interestingly, while RARG mRNA and protein levels had recovered 8 h after the final UVB treatment, RXRA mRNA and protein levels remained low. This suggests that RARG is refractory to UVB irradiation, where RXRA is not. However, protein levels of RXRs are normally five times that of RARs in human skin [97]. After UVB irradiation, the 1:1 ratio between RARG and RXRA leads to their heterodimerization to regulate transcription in the skin [97]. Andersson et al. (2003) examined the effects of UVB (50 mJ/cm^2^) on nuclear retinoid receptor levels in vitro [98]. Both keratinocytes and melanocytes experienced a rapid decline in RARA, RARG, and RXRG mRNA, and protein expression levels post-irradiation, but melanocytes experienced a full recovery within 1 to 3 days. In keratinocytes, however, only RARA had a complete recovery at 48 h post-irradiation.

In summary, UV irradiation significantly affects the retinoid levels in the epidermis and epidermal skin cells in vivo. In the epidermis, ultraviolet irradiation reduces retinol, retinyl esters, RXRA, RARA, and RARG. In contrast, UV irradiation increased RBP1, DHRS9, ALDH1A2, and CYP26A1. UVR did not affect ARAT or retinyl ester hydrolases. In cultured cells, keratinocytes and melanocytes have a rapid drop in RARA, RARG, and RXRG protein and mRNA levels. Melanocytes recover fully, but keratinocytes only experienced a full recovery of RARA 48 h after irradiation. Future studies should continue to examine the impact of UVR on retinoids in the skin and determine what mechanisms restore retinoid metabolism in the epidermis. These changes in retinoid metabolism may affect how skin recovers following UV irradiation. When skin does not fully recover, skin cancers develop.

## 8. Summary and Conclusions

In summary, research demonstrably argues that retinoids perform important regulatory roles in the hair and skin. Recent studies suggest that RA regulates the induction of anagen by inhibiting HFSCs, maintaining them in refractory telogen. RA also regulates catagen induction by a different mechanism. In addition, RA synthesized in HFSCs in vivo has been shown to induce the differentiation of McSCs. Similar increased differentiation was also seen if RA was provided early in that process in vitro. However, addition of RA to a fully differentiated melanocyte stunts that growth and causes the loss of their dendrites. In epidermal phototransduction, retinal is critical for UV-induced melanin synthesis, yet ultraviolet irradiation depletes both retinoids and their nuclear receptors, yet increases RA synthesis enzymes in the epidermis. Retinoid metabolism in the keratinocyte is well characterized. However, future studies are needed to determine both the specific retinoid metabolon in the melanocyte and the source of the retinal needed for phototransduction.

## Data Availability

Not applicable.
